# Geomorphic response to sea level and climate changes during Late Quaternary in a humid tropical coastline: Terrain evolution model from Southwest India

**DOI:** 10.1371/journal.pone.0176775

**Published:** 2017-05-03

**Authors:** Maya K., Vishnu Mohan S., Ruta B. Limaye, Damodaran Padmalal, Navnith K. P. Kumaran

**Affiliations:** 1 National Centre for Earth Science Studies, Thuruvaikkal P O, Thiruvananthapuram, Kerala, India; 2 Biodiversity and Palaeobiology Group, Palynology and Palaeoclimate Laboratory, Agharkar Research Institute, Pune, Maharashtra, India; Institute of Botany Chinese Academy of Sciences, CHINA

## Abstract

The coastal lands of southern Kerala, SW India in the vicinity of Achankovil and Thenmala Shear Zones reveal a unique set of geomorphic features like beach ridges, runnels, chain of wetlands, lakes, estuaries, etc. The chain of wetlands and water bodies that are seen in the eastern periphery of the coastal lands indicates the remnants of the upper drainage channels of the previously existed coastal plain rivers of Late Pleistocene age that are later broadened due to coastal erosion under the transgressive phase. The terrain evolutionary model developed from the results of the study shows that the Late Pleistocene transgressive events might have carved out a major portion of the land areas drained by the coastal plain rivers and as a result the coastal cliff has been retreated several kilometers landwards. The NNE—SSW trending beach ridges located close to the inland wetlands indicate the extent of shoreline shift towards eastwards during Late Pleistocene period. The present beach parallel ridges in the younger coastal plain indicate the limit of the Mid Holocene shoreline as the transgression was not so severe compared to Late Pleistocene event. The zone of convergence of the two sets of beach ridges coincides with the areas of economically viable heavy mineral placers that resulted from the size and density based sorting under the repeated transgressive events to which the coast had subjected to. The chain of wetlands in the eastern side of the study area has been evolved from a mega lagoon existed during Late Pleistocene. The Pallikkal River that links discrete eastern wetland bodies has been evolved into its present form during Early Holocene.

## 1. Introduction

Kerala coast in southern tip of India is known for its scenic and natural beauty as it is endowed with a unique association of different landform features of Late Quaternary origin and is a major depocenter of economically viable placer mineral resources. Like many of the world’s coastlines, the Indian coastline too has been modified due to coastal processes, climate changes and other geoevents including neotectonic activities in the Quaternary period [1–2]. The coastline has oscillated many times due to marine transgression and regression during the Late Quaternary period especially since Last Glacial Maximum [3–8]. Further, this humid tropical coastline has been subjected to environmental dynamics of the past several thousand years that eventually led to the formation of a wide range of geomorphologic features like bays, estuaries, deltas, marshes, dunes and beaches. The wetlands constitute one of the geomorphic features of this coastal state and it has three “Ramsar convention” designated sites—Vembanad, Ashtamudi and Sasthamkotta lakes. The origin, development and evolution of these wetlands have been addressed in recent years [7–10]. However, aspects related to the present day landscape and environment along with potential mineral resources, which are the products of Quaternary geological processes, caused by climate variations, sea level changes and tectonics are seldom looked into. In order to address it we have taken up the sediment archives retrieved from the subsurface coastal deposits of the uplifted block of South Kerala Sedimentary Basin (SKSB) between Achankovil and Thenmala Shear zones in the Trivandrum block as a case study site.

The study area is located at the extreme southern tip of the Indian subcontinent lying near the centre of the Indian tectonic plate. The coastal stretch is unique along west coast of India having a major depocenter with sediment fill of ~ 700 m thickness due to the presence of curvilinear landward extension of the offshore sedimentary basin referred to South Kerala Sedimentary Basin (SKSB).This landward extension, besides the older sediments, has developed about 80.0 m of relatively young sediments covering an age span of < 90,000 years to present and as such the signatures of Late Pleistocene and Holocene geological processes could be decoded while addressing the major theme of the contribution and proposing a terrain evolution model of the coastal lands.

The Quaternary sequence along west coast of India is better preserved in the SKSB in the southwestern part of India. SKSB is a landward extension of Kerala Konkan Basin (KKB) formed in this part of the southwest coast which owes its origin to the tectonics and collision of India-Eurasia plates. These latest regional tectonic elements are superimposed on the pre-existing trends creating a complex structural pattern affecting the drainage, landscape and depositional environments. In fact, the structural pattern seen in the Quaternary succession is largely inherited from the Neogene [6, 11] and this is the only area south of the Narmada, rift where the land area has subsided as the shelf and as such the Neogene-Quaternary sedimentary stratigraphy can be better studied from borehole cores of land. Major part of the basin is under a cover of lagoons, estuaries, perennial and seasonal wetlands, alluvial plains and the well known ridge-runnel systems and as such retrieval of subsurface sediments is essentially depended on borehole cores.

Among the three physiographic zones of Kerala, highland, midland and lowland, the latter apparently coincides with the coastal lands, covering about 10% of its total area. Apart from the geological importance, the coastal lands play a pivotal role in the socio-economic well being of the people in the State. The region is known for many economically viable mineral resources like beach placers, glass sand, lime shells, tile and brick clays, construction grade sand, etc., all of which are associated with the Quaternary deposits [12–13]. Further, the Quaternary sediments in the coastal plain act as a major aquifer system in the region. Irrespective of its socio-economic bearing, adequate studies in regard to its palaeoclimate and palaeoenvironmental potential have not been carried out in the area until 1980 [14]. One of the pioneer studies towards this direction was that of Nair [15]. He has made a detailed study on the geomorphologic and the Quaternary geological aspects of the coastal plains of Kannur and Kasaragod districts. Mathai and Nair [16] investigated the emergence of the west coast taking an example from central Kerala. Nair [17] extended the study on the geomorphology and the evolution of coastal landforms all along the Kerala coast. The major breakthrough in Kerala Quaternary studies was achieved during the last decade [8–10, 18–21]. Further, contributions of Thrivikramji and Anirudhan [22], Nair [23], Narayana et al. [24], Joseph and Thrivikramji [25], Narayana [26], Narayana and Priju [27], Nair [28] and Thrivikramji et al. [29] have helped immensely to unfold the different aspects of the Quaternary geology of Kerala coast.

Although many studies on the Quaternary geology of the South Kerala Sedimentary Basin have been attempted, adequate attention has not been given to the coastal triangular area of the Trivandrum block between the Pallikkal and Achankovil rivers in Southern Kerala which is characterized by unique geomorphic features. Some of the important observations are presence of a series of wetlands bordering the coastal area, two sets of beach ridges intersecting at an angle, occurrence of heavy mineral placers in the beaches and certain beach ridges, proneness to Tsunamis and other storm surges. The coastal triangular area is bounded by two lineaments, *viz*., the Achankovil and Thenmala. In view of having these unique landforms in this block, which were evolved and developed, as a consequence of sea level changes and the coastal processes, the present communication has been focused in the form of an evolutionary model. The sediment archives retrieved from the subsurface coastal deposits of the uplifted block of South Kerala Sedimentary Basin between Achankovil and Thenmala Shear zones in the Trivandrum block have provided the geomorphic signatures. These signatures are decoded with the help of sedimentological, palynological and geochronogical data which in turn helped addressing aspects related to evolution of landform units. The landform dynamics in response to sedimentation pattern and development of various geomorphic units prior to Late Pleistocene to the present has been provided in the form a terrain evolution model.

## 2. Study area

**Fig 1:**
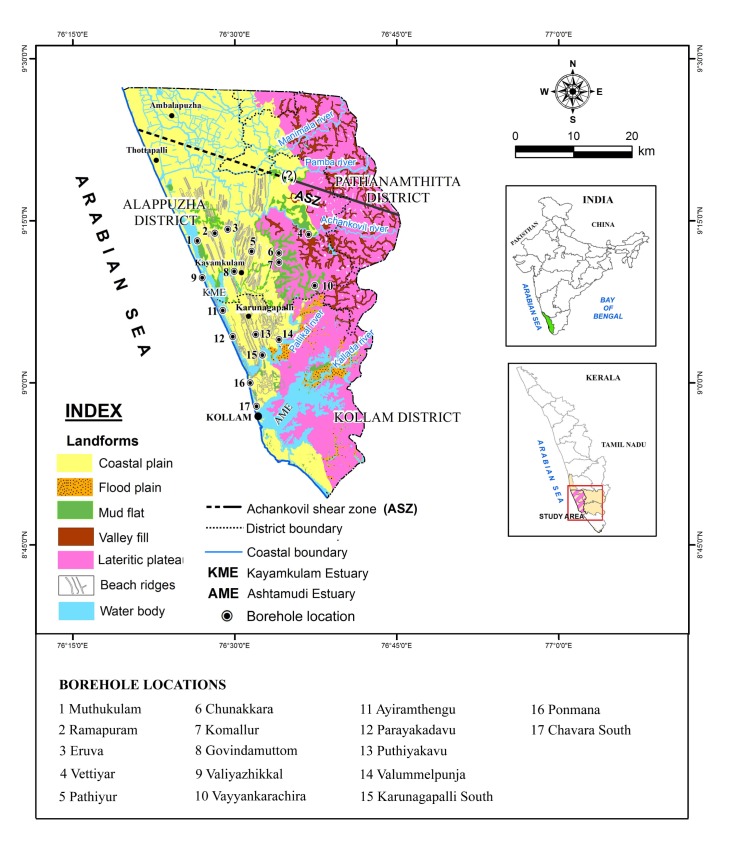
Major landform features of the study area showing location of borehole cores.

The study area falls within the coastal lowlands of southern Kerala (SW India) between Achankovil and Pallikkal rivers. The area comprises three major rock types/lithological units: 1) the Precambrian crystallines, 2) Neogene sediments and 3) Late Quaternary deposits. The Precambrian crystalline basements of southern Kerala is dominated by high-grade metamorphic rocks viz; khondalite (garnet-sillimanite gneiss) and charnockite (pyroxene granulite) suites of rock. This area is marked by two prominent lineaments trending NW–SE, the southern one is marked by the Kallada River, whereas the northern counterpart is marked by the Achankovil River. In the highlands, these two rivers are almost parallel and coincide with the respective lineaments. On the contrary, in the midlands the channels diverge—the Kallada River takes a WSW course while the Achankovil River takes a NNW course. The triangular area bounded by these two rivers is drained by a lowland river—the Pallikkal River which originates from Adoor, joining the southern arm of the Kayamkulam lagoon near Vatta *kayal*. One of the prominent geomorphic features of the triangular area is the occurrence of a series of wetlands in its eastern end (Fig 1). The downstream margin of these wetlands truncates against a set of palaeo-beach ridges. Preliminary investigations of these wetlands indicate that they are composed essentially of black, stiff clay at the top which often embed uprooted plants/wood [18].

## 3. Material and methods

**Fig 2:**
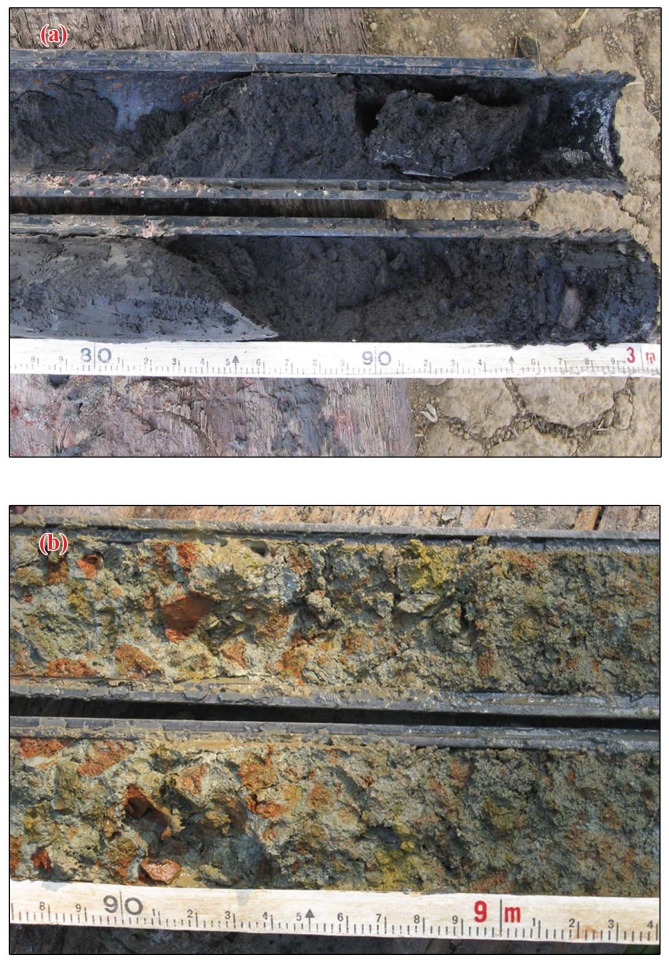
a) Peat deposit in a borehole core retrieved from Vettiyar site; b) Ferruginous conglomerate with pebbles from laterised provenance.

Prior permission for field work and drilling of boreholes in the study area was obtained from the Directors of National Centre for Earth Science Studies, Thiruvananthapuram and Agharkar Research Institute, Pune, India. Accordingly, no specific permissions were required to carry out field work and drilling activities in the locations selected for our study. It is also hereby confirmed that the field studies did not involve any endangered or protected species. Systematic fieldwork was carried out in the coastal stretch between Kallada and Achankovil rivers for the collection of data on various landform features and also for locating borehole sites for subsurface sample collection. Mapping of the wetlands and related landform features has been carried out in 1:25000 scales. A total of 17 borehole core sediments (Fig 1) were collected using rotary drilling unit fitted with Split Sediment Sampler—a method widely used for subsurface sample collection [7,9–10]. The uncontaminated subsurface samples and their lithological features of the split borehole cores are shown in Fig 2. Out of the 17 borehole cores, 8 are from younger coastal plains, 3 from older coastal plains and 6 from the wetlands/palaeoestuaries bordering old coastal plains. After documenting the gross lithological details, the borehole cores were sectioned at 10.0 cm intervals and sub-samples from selected depths were packed in neatly labeled polythene bags for further laboratory analysis. Utmost care was taken in the field itself to avoid contamination during sub-sampling, packing and processing for various analytical procedures.

The sediment samples were subjected to textural analysis following Lewis [30]. The ternary diagram of Picard [31] was used for classification of sediments. The organic carbon (C_-org_) contents of the sediments were determined following the wet oxidation method of El Wakeel and Riley [32], whereas carbonate carbon (C_-inorg_) contents by titrimetric method of Jackson [33]. Average of triplicate measurements not differing 0.2% of the analyses was used in this study. Selected samples of the organic matter rich sediments were subjected to palynological examinations. The samples for recovering palynomorphs were processed by conventional method of separating organic walled microfossils from sediments [34–35]. Photomicrographs were taken in a Canon Powershot digital camera. Radiocarbon (^14^C) dates of a few samples of subfossil wood and sediments at specific levels were determined at Birbal Sahni Institute of Palaeobotany, Lucknow (India) and these dates are non- calibrated ages.

## 4. Results

The coastal Quaternary sediments register records of past climate and sea level changes.

Although many attempts have been made while addressing these changes [9, 28, 36–40], no systematic effort has been made in the sedimentary archives of southwest coast of India, especially in the northwestern part of the Trivandrum block between Achankovil and Thenmala lineaments/shear zones [41]. This section presents the results of the investigations carried out in the subsurface and surface sediments of the coastal lands between the Achankovil and Thenmala lineaments drained by the Pallikkal River.

### 4.1. Lithology and sediment characteristics

**Fig 3:**
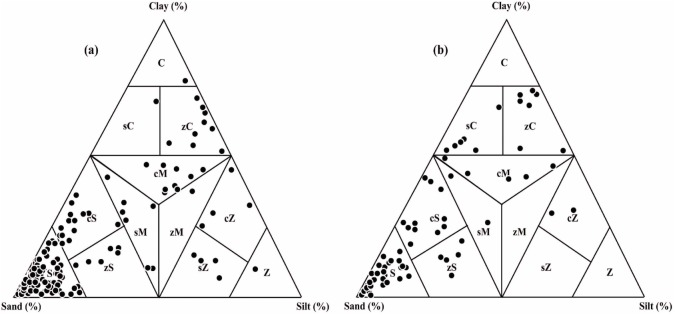
Sediment types (after Picard [31]) of the borehole samples retrieved from coastal plains (a) and wetlands (b) of the study area.

**Fig 4:**
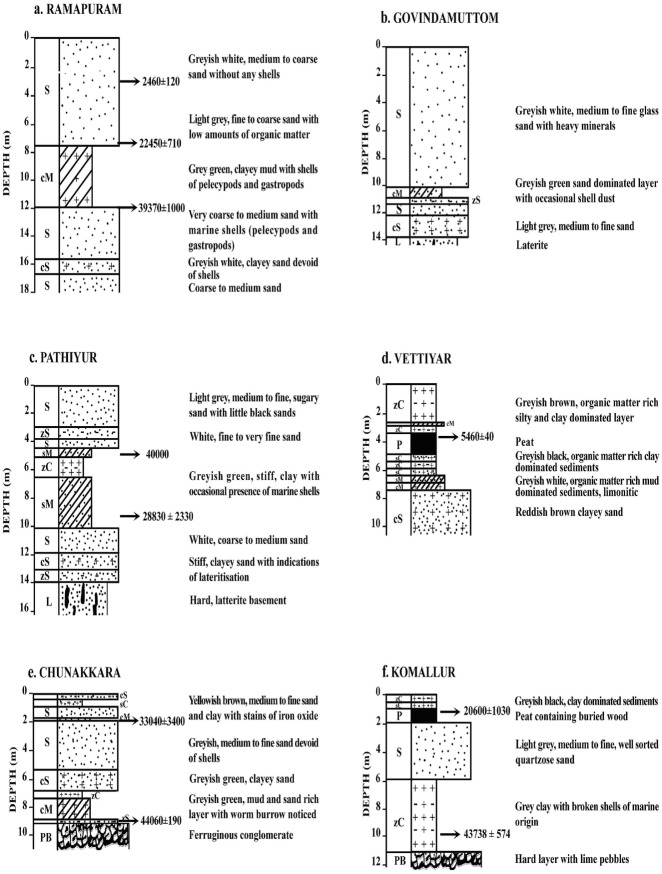
Lithological characteristics of (a) Ramapuram, (b) Govindamuttom, (c) Pathiyur, (d) Vettiyar, (e) Chunakkara and (f) Komallur borehole cores. S Sand, zS Silty sand, cS Clayey sand, sM Sandy mud, cZ Clayey silt, zM Silty mud, cM Clayey mud, sC Sandy clay, zC Silty clay, L Laterite, P Peat, PB Pebble bed.

Fig 3 shows the sediment types of some selected borehole cores retrieved from the study area. As seen from this figure, sand is the dominant sediment type in most of the borehole samples. It is followed by clayey sand, silty clay, sandy mud, clayey mud, silty sand, sandy silt, silt and clay in the decreasing order. Silt dominant sediment species are lesser in number in the wetlands than the coastal plains. The lithological characteristics of the borehole cores retrieved from different landform features of the study area are depicted in Table 1. The borehole cores retrieved from the younger coastal plains such as Muthukulam, Ramapuram, Valiyazhikkal, Ayiramthengu, Parayakadavu, Ponmana, Chavara South and Govindamuttom are composed essentially of sand dominant sediments with variable thickness (1.0–10.0 m) at the top followed by silt and clay dominated layer in most of the cores, except Ponmana and Chavara South borehole cores. The mud dominated layer of the Muthukulam core embeds tree trunks and sub-fossil logs in the lower part. Occasional presence of shells is also noticed in the sediments. The organic carbon content varies from 0.7% to 5.2% [42]. The mud dominated sediments seen below the sand apron at Ramapuram is rich in organic matter and often contains presence of pelecypod and gastropod shells of marine affinity (Fig 4A). The upper part of the bottom sand also embeds many broken and unbroken shells (Table 1). The Valiyazhikkal borehole core, retrieved from the littoral zones, is composed essentially of sand dominant sediments followed downward by clayey mud (2.0 m), sand (1.5m), muddy sand (4.5m), sand (5.25m) and muddy sand (> 2.5m). The coarser particles at the bottom show the presence of eroded materials derived mainly from lateritized terrains. Ayiramthengu borehole is sited on the south eastern bank of the Kayamkulam lagoon. The sediment column is intervened at two levels (9.8–11.0 m bgl and 17.0–22.0 m bgl) by organic matter rich, silt and clay dominated sediments. The top 9.5m thick, coarse to medium grained sand records occasional presence of molluscan (gastropod and pelecypods) shells. On an average, the borehole core contains 93.99% (89.53–97.62%) sand, 2.36% (1.20–4.64%) silt and 3.70% (0.75–7.59%) clay (Table 2). The sands layer rests over 1m thick, stiff, greyish green clayey mud. The lithological unit occurring below the clayey mud is a 6.0 m thick white sand layer. It is followed downward by 5.0 m thick, stiff, silt and clay dominated sediments (1.0 m thick clayey silt and 4m thick silty clay). The litho sequences rests over sand dominated, lateritized sediment. Hard laterite (duricrust?) occurs at about 25.0 m below ground level. The borehole core at Parayakadavu comprises three distinct lithounits. The top 6.5 m represents a sand layer which is followed downward by 2.5 m thick sandy silt with shells of pelecypods and gastropods. The 10.0 m long borehole core retrieved from Ponmana is composed essentially of greyish brown sand dominated sediments which rest over lateritic basements. The sand, silt and clay contents of the upper sand layer are 87.33% to 97.83% (av. 92.58%), 1.01% to 3.74% (av. 2.38%) and 1.16% to 8.93% (av. 5.05%), respectively (Table 2). The textural analysis of the borehole core at Chavara south is composed of sand dominant sediments which rest over a hard laterite basement. Textural analysis reveals that the upper half of the sand layer is made up of light grey sand, whereas the lower half is made up of light yellow sand. The sand, silt and clay contents in the sand dominated layer are 80.46% to 93% (av. 87.31%), 2.55% to 10.94% (av. 5.51%) and 4.07% to 12.33% (av. 7.18%), respectively (Table 2). The Govindamuttom borehole core is retrieved from a site located about 2.0 km southwest of Kayamkulam town. The Govindamuttom core is composed essentially of sand dominated sediments (Fig 4B). The greyish white, sand layer (~10.2m) at the top contains 82.37% to 93.70% (av. 89.70%) of sand, 0.08% to 4.04% (av. 1.37%) of silt and 6.22% to 14.42% (av. 8.93%) of clay. The underlying layer is 1m thick, greyish green clayey mud with sand, silt and clay contents of 49.01% to 49.96% (av. 49.49%), 21.06% to 22.84% (av. 21.95%) and 28.15% to 28.98% (av. 28.57%), respectively (Table 2). This layer shows occasional presence of shell dusts which is followed by a sandy layer (60.88%– 79.11% of sand; 7.79%– 14.61% of silt and 11.20%– 28.98% of clay fractions). The entire sequence lies unconformably over a wormiform laterite.

**Table 1:** Lithological characteristics of the borehole cores retrieved from different landform features of the study area.

Landforms/borehole cores	Borehole length (m)	Major observations
**I—Younger coastal plain**		
1. Muthukulam	3	The borehole core composed essentially of 1.0 m thick sand dominant sediments followed by clayey silt (1.5 m thick) and silty clay (>0.5m thick). This layer embeds tree trunks and subfossil logs in its lower part. Occasional presence of shells is also noticed in the silt and clays dominated sediments. The sediments are of Holocene in age. The organic carbon content varies from 0.7% to 5.2%.
2. Ramapuram	18	The borehole core composed mainly of a sand dominated layer which is intervened at the middle (7.5–12.0 m) by clayey with occasional presence of pelecypod and gastropod shells of marine affinity. The upper part of the bottom sand contains many broken and unbroken shells.
3. Valiyazhikkal	20	The borehole core is retrieved from the littoral zone of the study area and composed essentially of sand dominant sediments. The borehole core begins with a 4.0 m thick sand layer followed downward by 2.0 m thick clayey mud, 1.5m thick sand, 4.5m thick muddy sand, 5.25m thick sand and > 2.5m thick muddy sand. The coarser particles at the bottom show indications of eroded materials derived from lateritised terrain.
4. Ayiramthengu	26	The borehole is sited on the south eastern bank of the Kayamkulam lagoon and composed essentially of sand dominant sediments. The sediment column is intervened at two levels (9.8-11m bgl and 17-22m bgl) by organic matter rich, silt and clay dominated sediments. The top 9.5m thick, coarse to medium grained sand records occasional presence of molluscan (gastropod and pelecypod) shells. The sands layer rests over 1.0 m thick, stiff, greyish green clayey mud. The lithologic unit occurring below the clayey mud is a 6.0 m thick white sand layer. It is followed downward by 5.0 m thick, stiff, silt and clay dominated sediments (1.0 m thick clayey silt and 4.0 m thick silty clay). The litho sequences rests over sand dominated, lateritized sediment. Hard laterite (duricrust?) occurs at about 25.0 m below ground level (bgl).
5. Parayakadavu	14	The borehole core comprises three distinct lithounits. The top 6.5 m represents a sand layer which is followed downward by 2.5 m thick sandy silt with shells of pelecypods and gastropods.
6. Ponmana	10	The borehole core composed essentially of greyish brown sand dominated sediments which rest over lateritic basements. The sands are devoid of shells and other calcareous materials.
7. Chavara South	8	The upper 6m thick borehole core is composed mainly of sand dominant sediments which rests over a hard laterite basement. Textural analysis reveals that the upper half of the sand layer is made up of light grey, medium to fine sand, whereas the lower half is made up of light yellow sand.
8. Govindamuttom	14	The borehole core is retrieved from a site located about 2.0 km southwest of Kayamkulam town and core is made up essentially of sand dominated sediments. The greyish white, sand layer (~10.2m) at the top ifs followed by 1.0 m thick, greyish green clayey mud. This layer shows occasional presence of shell dusts. The entire sequence lies unconformably over a wormiform laterite basement.
**II—Older coastal plain**		
1. Puthiyakavu	6	The borehole core is composed of 3.0 m thick sand resting over a laterite basement. The sands are free from shells and other calcareous material.
2. Pathiyur	16	The borehole is sited on the bank of a minor channel draining the wetlands on the eastern side of the Kayamkulam lagoon. The borehole composed of three major lithounits– 4.2m thick, light grey sandy sediments at the top followed by 5.8m thick, greyish green mud dominated sediments and 4.0 m thick, light grey sand dominated sediments towards bottom. The entire sequence rests over a hard laterite basement. The middle mud dominated layer contains broken and unbroken molluscan shells of pelecypods and gastropods.
3. Eruva	8	The borehole core at Eruva is dominated by sand and silt dominated sediments upto a depth of 5.5 m. This layer rests over organic rich grey clay. The middle and lower part of the borehole core contains occasional presence of broken and unbroken pelecypods and gastropods. The topmost part of the sediment column is made up of 1.0 m thick sand stratum. The bottom clay is organic matter rich and is dark greenish to olive grey in colour.
III Wetlands		
1.Vettiyar	10	The borehole core composed of 7.5m thick silt and clay dominated sediments followed by 2.0 m thick sand dominated sediments. The top clay dominated layer is intervened at 3.4–4.9m level by 1.5m thick peat layer. The 2.5m thick top layer of the borehole core is dominated by silt and very fine clay indicating a floodplain sequence.
2. Karunagapalli south	14	The borehole core generally composed of sand dominated sediments. The sand layer is intervened at 0.9–1.9m level by ~1.0 m thick mud dominated sediments.
3. Chunakkara	10	The borehole core composed of 7.0 m thick sand dominated sediments at the top followed by ~2.3 m thick mud dominated sediments. The sand dominated sequence shows occasional presence of broken shells/ shell dusts. This layer is intervened by mud dominated layers. The sequence rests unconformably over a ferruginous conglomerate whose upper surface is erosional with pebbles derived from lateritic terrains. The top sand layer of the Chunakkara borehole core exhibits a coarsening upward sequence with dominance of medium and fine sands at the top and, fine and very fine sands in the rest of the core. Even though the top layer of the borehole core is dominated by fine and very fine sands, very fine clay also occurs in appreciable amounts. The sand layer is followed by silty clay and clayey mud with dominance of coarse and fine clay.
4. Komallur	12	The borehole core composed of 1.0 m thick clay dominated sediments at the top followed by 4.0 m thick sandy sediments and 5.0 m thick clayey sediments at the bottom. The top clay and sand layers are intervened by 1.0 m thick peat.
5. Valummelpunja	8	The borehole composed of white coloured silica sand at the top (3.9m thick) and greyish white, clayey sand at the bottom (2.0 m thick) with an interlayer of clay dominated sediments at 3.9–4.8m level. The entire sequence lies unconformably over a ferruginous conglomerate. The top 4.0 m thick sand deposit of the core exhibits dominance of medium, fine and very fine sands. The bottom 2.5m thick sand layer is dominated by coarse, medium and fine sand with traces of very fine gravel.
6. Vayyankarachira	0.8	The borehole core composed essentially of clay dominated upper layer (0.4m thick) and a mud dominated (0.4–0.8m bgl) lower layer.

**Table 2:** Textural characteristics of the borehole cores[Depth range is given in parenthesis].

Borehole Location	Sediment type/ Depth range (m)	Sand (%)			Silt (%)			Clay (%)		
		Min.	Max.	Av.	Min.	Max.	Av.	Min.	Max.	Av.
1. Valiyazhikkal	Sand (0–4)	78.12	93.52	87.82	4.53	22.00	10.17	0.12	3.57	2.05
	Clayey mud (4–6)	25.53	29.32	27.43	31.52	36.26	33.89	38.21	39.16	38.69
	Sand (8–20)	76.52	93.45	85.11	2.09	20.12	9.75	1.40	10.58	5.14
2. Ramapuram	Sand (0–7.5)	87.70	97.90	92.66	1.10	5.00	2.70	1.00	11.00	5.38
	Clayey mud (7.5–12)	12.00	29.70	21.02	31.40	41.20	35.48	38.10	46.90	43.36
	Sand & clayey sand (12–18)	64.00	97.70	85.08	1.20	5.00	2.80	1.10	33.60	12.55
3. Ayiramthengu	Sand (0–9.5)	97.28	97.62	95.03	1.20	1.98	1.63	0.75	3.26	2.17
	Clayey mud(9.5–10.5)	31.49	31.49	31.49	20.80	20.80	20.80	47.71	47.71	47.71
	Sand (10.5–12.5)	89.53	89.94	89.76	2.88	4.64	3.48	7.28	7.59	7.43
	Clayey silt & silty clay (12.5–19.5)	0.23	4.21	2.22	32.80	64.51	48.66	35.26	62.99	49.13
	Silty & clayey sand (19.5–26)	51.78	56.35	54.07	23.63	26.90	25.27	16.75	24.59	20.67
4. Parayakadavu	Sand (0–6.5)	80.23	97.21	90.01	0.90	12.34	6.32	1.68	7.72	3.68
	Sandy silt (6.5–9)	25.02	31.25	28.75	53.21	61.25	56.90	13.73	15.54	14.35
	Sand (9–14)	78.21	94.56	85.85	1.21	18.21	11.35	2.80	1.71	18.24
5. Ponmana	Sand (0–1.5)	87.33	97.83	92.58	1.01	3.74	2.38	1.16	8.93	5.05
6. Chavara South	Sand (0–6)	80.46	93.00	87.20	2.55	10.94	5.74	4.07	12.33	7.36
7. Puthiyakavu	Clayey sand (0–3)	68.64	69.39	69.02	5.12	10.16	7.64	20.45	26.24	23.35
	Laterite (3–7)	Not analyzed								
8.Govindamuttom	Sand (0–10.2)	82.37	93.70	88.04	0.08	4.04	2.06	6.22	14.42	10.32
	Sandy clay (10.2–10.9)	53.01	54.96	53.99	20.06	21.84	20.95	24.98	25.15	25.07
	Silty sand & sand (10.9–12.2)	74.19	79.11	76.65	8.00	9.67	8.84	11.20	11.22	11.21
	Clayey sand (12.2–13.9)	60.88	70.99	65.94	7.79	10.14	8.97	21.11	28.98	25.05
	Conglomerate (13.9–14)	Not analyzed								
9. Pathiyur	Sand & silty sand (0–4.5)	55.04	89.24	77.38	1.21	26.38	7.42	9.55	19.21	15.20
	Sandy mud & silty clay (4.5–10)	1.15	49.06	28.53	19.65	39.25	31.91	11.69	61.53	39.56
	Sand, clayey & silty sand (10–14)	58.19	86.30	68.35	2.04	23.83	10.23	8.88	37.41	21.42
	Laterite (14–16)	Not analyzed								
10. Vettiyar	Silty clay (0–2.7)	17.99	17.99	17.99	28.06	28.06	28.06	53.95	53.95	53.95
	Clayey mud (2.7–2.9)	20.74	20.74	20.74	35.63	35.63	35.63	43.63	43.63	43.63
	Silty clay (2.9–3.4)	1.89	1.89	1.89	24.4	24.4	24.4	73.71	73.71	73.71
	Peat (3.4–4.9)	Not analyzed								
	Sandy clay (4.9–6.4)	34.23	44.25	39.24	2.57	13.35	7.96	52.42	56.69	54.56
	Sandy & clayey mud (6.4–7.4)	46.78	47.38	47.08	3.79	9.75	6.77	43.47	48.83	46.15
	Clayey sand (7.4–9.7)	54.63	57.9	56.27	0.52	16.96	8.74	25.14	43.43	34.29
11. Karunagappalli South	Sand (0.8–0.9)	91.52	94.28	92.90	0.08	2.34	1.21	5.64	6.14	5.89
	Clayey mud (0.9–1.9)	18.03	18.03	18.03	40.11	40.11	40.11	41.86	41.86	41.86
	Sand (1.9–9.4)	86.27	91.86	89.07	0.14	7.31	3.73	6.42	11.22	8.82
	Clayey sand (9.4–11.4)	71.68	71.68	71.68	2.87	2.87	2.87	25.45	25.45	25.45
	Sand (11.4–14)	81.24	89.45	85.35	1.07	2.40	1.74	8.15	16.96	12.56
12. Komallur	Silty & sandy clay (0–1)	6.74	16.79	11.77	15.10	24.20	19.65	68.11	69.06	68.59
	Peat (1–2)	Not analyzed								
	Sand (2–6)	96.04	99.01	98.01	0.28	1.90	1.00	0.19	2.06	0.81
	Silty clay (6–11)	2.77	2.97	2.87	22.80	23.99	23.40	73.24	74.23	73.74
	Conglomerate (11–12)	Not analyzed								
13. Chunakkara	Clayey sand (0–0.4)	70.73	70.73	70.73	2.32	2.32	2.32	26.95	26.95	26.95
	Sandy clay (0.4–0.9)	37.43	37.43	37.43	7.77	7.77	7.77	54.80	54.80	54.80
	Sand (1.4–1.8)	83.71	95.71	89.71	0.33	2.07	1.20	2.22	14.54	8.38
	Clayey mud (1.8–1.9)	41.56	41.56	41.56	14.66	14.66	14.66	43.77	43.77	43.77
	Sand (1.9–5.4)	91.51	96.08	93.80	0.11	0.72	0.42	1.99	7.77	4.88
	Clayey sand (5.4–6.9)	61.19	61.19	61.19	15.42	15.42	15.42	23.46	23.46	23.46
	Silty clay (6.9–7.4)	4.58	4.58	4.58	45.14	45.14	45.14	50.28	50.28	50.28
	Clayey mud & silty sand (7.4–9.2)	8.29	53.50	30.90	25.51	45.77	35.64	21.01	45.94	33.48
	Conglomerate (9.2–11)	Not analyzed								
14.Valummelpunja	Sand (0–3.9)	85.79	89.97	87.88	0.72	6.28	3.5	7.93	12.41	10.17
	Silty clay (3.9–4.8)	8.56	9.27	8.92	18.25	19.6	18.93	71.14	73.19	72.17
	Sand & silty sand (4.8–5.4)	63.33	84.79	74.06	3.67	21.48	12.58	11.54	15.19	13.37
	Clayey sand (5.4–7.5)	66.57	68.76	67.67	5.42	6.71	6.07	25.47	27.10	26.29
	Conglomerate (7.5–8)	Not analyzed								

The older coastal plain seen at the eastern half is quite different from the younger coastal plains in the western side. The most notable feature is the difference in the orientation of the ridge—runnel system. While the ridges and runnels show an orientation of NNE-SSW in the older coastal plains, the ridge runnel system in the younger coastal plains are quite different and are almost parallel to the present coastal line. The borehole cores retrieved from Puthiyakavu, Pathiyur and Eruva fall under this category. The Puthiyakavu core is composed of 3m thick sand resting over a laterite basement. The sand, silt and clay contents of the layer are 68.64% to 69.39% (av. 69.02%), 5.12% to 10.16% (av. 7.64%) and 20.45% to 26.24% (av. 23.35%), respectively (Table 2). The Pathiyur borehole is located on the bank of a minor channel draining the wetlands in the eastern side of the Kayamkulam lagoon. The core is consists of three major lithounits—4.2 m thick, light grey sandy sediments at the top followed by 5.8 m thick, greyish green mud dominated sediments and 4m thick, light grey sand dominated sediments towards bottom (Fig 4C). The respective proportion of sand, silt and clay in the upper sandy layer are 55.04% to 89.24% (av. 77.38%), 1.21% to 26.38% (av. 7.42%) and 9.55% to 19.21% (av. 15.20%), while that in the middle mud dominated layer are 1.15% to 49.06% (av. 28.53%), 19.65% to 39.25% (av. 31.91%) and 11.69% to 61.53% (av. 39.56%). The bottom sand dominated layer accounts for 58.19%– 86.30% (av. 68.35%) of sand, 2.04%– 23.83% (av. 10.23%) of silt and 8.88%– 37.41% (av. 21.42%) of clay (Table 2). The entire sequence rests over a hard laterite basement. The middle mud dominated layer contains broken and unbroken molluscan shells of pelecypods and gastropods. The borehole core of Eruva is dominated by sand and silt upto a depth of 5.5 m. This layer rests over organic rich grey clay. The middle and lower part of the borehole core contains occasional presence of broken and unbroken pelecypods and gastropods. The topmost part of the sediment column is made up of 1.0 m thick sand stratum. The bottom clay is organic matter rich and is dark greenish to olive grey in colour.

A total of six borehole cores are collected from wetlands or areas close to it. Most of the wetlands in the region are used for paddy cultivation. Out of the six cores, the ones retrieved from Vettiyar and Karunagapalli south are located respectively in the floodplains/over bank areas of the Pallikkal and Achankovil rivers. The other cores are retrieved from wetlands in the eastern periphery of the study area. These borehole cores include the cores collected from Chunakkara, Komallur, Vayyankarachira and Valummelpunja. The Vettiyar core is comprised of 7.5 m thick silt and clay dominated sediments followed by 2.0 m thick sand dominated sediments (Fig 4D). The top clay dominated layer is intervened at 3.4–4.9 m level by 1.5 m thick peat layer. The 2.5 m thick top layer of the borehole core is dominated by silt and very fine clay indicating a floodplain sequence. In the Vettiyar core, the mean size of the silt and clay dominated sediments (7.5 m thick) varies from 6.447Ф (medium silt) to 8.518Ф (very fine silt). The sediments are poorly sorted (1.932Ф) to very poorly sorted (3.890Ф), symmetrical (0.072) to very coarse skewed (-0.662) and very platykurtic (0.664) to platykurtic (0.536). The sand, silt and clay contents of the Karunagapalli South borehole core is 71.68% to 94.28% (av. 87.43%), 0.08% to 7.31% (av. 2.23%) and 5.64% to 25.45% (av. 10.35%), respectively (Table 2). The sand layer is intervened at 0.9–1.9m level by ~1m thick mud dominated sediments. The sand dominated sequence shows occasional presence of broken shell and shell dusts in the Chunakkara borehole core (Fig 4E). The layer is interspersed by a couple of mud dominated thin layers. The sand, silt and clay contents in this sand dominated layer are 61.19% to 97.47% (av. 87.07%), 0.11% to 15.42% (av. 2.61%) and 1.99% to 26.95% (av. 10.33%), respectively whereas the same fractions in the mud dominated layer are 8.29% to 41.84% (av. 25.58%), 30.19% to 45.77% (av. 35.60%) and 27.31% to 45.94% (av. 38.81%), respectively (Table 2). The sequence rests unconformably over a ferruginous conglomerate whose upper surface is erosional with pebbles derived from lateritic terrains. The top sand layer of the Chunakkara core exhibits a coarsening upward sequence with dominance of medium and fine sands at the top and, fine and very fine sands in the rest of the core. Even though the top layer of the borehole core is dominated by fine and very fine sands, very fine clay also occurs in appreciable amounts. The sand layer is followed by silty clay and clayey mud with dominance of coarse and fine clay. The mean size of this 11.0 m thick core varies from 3.498Ф (very fine sand) to 6.505Ф (fine silt). All the samples are very poorly sorted (3.145Ф - 3.748Ф) and generally very fine skewed (0.651) to coarse skewed (-0.266). A few samples exhibit symmetrical skewness (0.024 to 0.078). Kurtosis varies from 0.602 (very platykurtic) to 1.018 (mesokurtic).

The top clay and sand dominated layers of the Komallur borehole core are intervened by 1.0 m thick peat (Fig 4F). The sand, silt and clay contents in the top sand dominated layer are 96.04% to 99.01% (av. 98.01%), 0.28% to 1.90% (av. 1.00%) and 0.19% to 2.06% (av. 0.81%), respectively and bottom clay dominated layer are 2.77% to 2.97% (av. 2.87%), 22.80% to 23.99% (av. 23.40%) and 73.24% to 74.23% (av. 73.74%), respectively (Table 2). The Valummelpunja wetland is comprised of white coloured silica sand at the top (3.9 m thick) and greyish white, clayey sand at the bottom (2.0 m thick) with an interlayer of clay dominated sediments at 3.9–4.8 m level. The entire sequence lies unconformably over a ferruginous conglomerate. The top 4.0 m thick sand deposit of the core exhibits dominance of medium, fine and very fine sands. The bottom 2.5 m thick sand layer is dominated by coarse, medium and fine sand with traces of very fine gravel. The mode of grain size population in the upper 4.0 m thick, sand dominated sediments vary from 2.638Ф to 2.708Ф. The samples are poorly sorted (1.795Ф to 1.867Ф), very fine skewed (0.320 to 0.330) and very leptokurtic (1.957 to 2.080). The middle, 1.0 m thick, silty clay layer shows a mean size of 8.817Ф - 2.554Ф. The samples are very coarse skewed (-0.772) and mesokurtic (0.930). The bottom 2.5m thick sand shows mean size range of 4.335Ф - 4.634Ф. The samples are very poorly sorted (3.992Ф) to extremely poorly sorted (4.037Ф), very fine skewed (0.538 to 0.599) and very platykurtic (0.599 to 0.652). The short core retrieved from Vayyankarachira is composed essentially of clay dominated upper layer (0.4 m thick) and a mud dominated (0.4–0.8m bgl) lower layer. The sand, silt and clay contents vary from 6.51% to 7.19%, 15.90% to 38.15% and 54.66% to 77%, respectively, in the clay dominated layers and 25.72% to 46.44%, 18.21% to 23.28% and 31.72% to 51.43% in the mud dominated layers. The content of clay in the upper half of the core shows an increasing trend. The top most sample is made up of 77.59% of clay, 15.90% of silt and 6.51% of sand.

The borehole cores retrieved from the coastal plains of Ramapuram, clayey mud (8.0 to 12.0 m depth bgl) exhibits the highest organic carbon content (2.13–2.58%). The clayey mud with shells of pelecypods and gastropods record the highest CaCO_3_ content (14.5–19.55%). The Chavara South core (av. 0.09%) record the lowest content of C_-org_ as the core is composed essentially of quartz rich sand dominated sediments. The silt and clay rich sediments occurring at the lower part of the Ayiramthengu borehole core show higher content of C_-org_ (av. 3.13%) than that of the upper sand dominant layers (av. 0.27%); (Table 3). A similar trend is noticed in the Karunagapalli South and Govindamuttom borehole cores as well. The respective ranges of C_-org_ are BDL to 16.20% (av. 5.69%) for the Karunagapalli South and BDL to 1.45% (av. 0.60%) for the Govindamuttom cores (Table 3). In the Govindamuttom core, the lowest level of organic carbon is recorded for the sand dominated sediments. The values of organic carbon (BDL– 47.63%; av. 1.46%) and carbonate carbon (BDL– 19.5%; av. 1.77%) exhibit wide variations in the Komallur borehole sediments. The anomalously high concentration of organic carbon (47.63%) is noticed for the peat layer occurring at a depth of 1.4 m from the top. With exception of this, all the other samples of the Komallur core yielded an average of 1.06% of organic carbon.

**Table 3:** Organic(C_-org_) and carbonate carbon(C_-inorg_) contents in the borehole cores. [BDL-Below Detection Limit; Depth range is given in parenthesis].

Sl. No.	Location	Sediment type/ Depth range (m)	C_-org_ (%)			C_-inorg_ (%)		
			Min.	Max.	Av.	Min.	Max.	Av.
1	Ramapuram	Sand (0–7.5)	0.14	0.25	0.20	BDL	0.50	0.17
		Clayey mud (7.5–12)	2.13	2.61	2.44	14.50	19.50	17.00
		Sand & clayey sand (2–18)	0.06	0.08	0.07	1.00	8.50	4.75
2	Ayiramthengu	Sand (0–9.5)	0.22	0.32	0.27	0.74	1.30	0.98
		Clayey mud (9.5–10.5)	3.13	3.13	3.13	0.68	0.68	0.68
		Sand (10.5–17)	0.07	0.07	0.07	1.00	1.00	1.00
		Clayey silt & silty clay (17–22)	0.95	1.38	1.16	0.93	0.99	0.96
		Silty sand and clayey sand (22–26)	0.37	0.37	0.37	1.74	1.74	1.74
3	Ponmana	Sand (0–1.5)	0.05	0.30	0.18	0.41	1.68	1.05
4	Chavara South	Sand (0–6)	BDL	0.23	0.09	0.12	1.34	0.81
5	Govindamuttom	Sand (0–10.2)	BDL	3.11	0.52	0.11	3.52	1.55
		Sandy mud (10.2–10.9)	BDL	1.45	0.72	0.23	1.78	1.00
		Silty sand & Sand (10.9–12.2)	0.79	1.05	0.92	0.70	1.63	1.16
		Clayey sand (12.2–13.9)	0.13	0.36	0.25	0.15	0.22	0.19
		Conglomerate (13.9–14)	Not analyzed					
6	Pathiyur	Sand & silty sand (0–4.5)	0.07	0.19	0.12	BDL	BDL	BDL
		Sandy mud & silty clay (4.5–10)	0.41	2.12	0.98	BDL	BDL	BDL
		Sand, clayey & silty sand (10–14)	0.12	0.31	0.22	BDL	BDL	BDL
		Laterite (14–16)	Not analyzed					
7	Vettiyar	Silty clay (0–2.7)	6.66	6.66	6.66	0.37	0.37	0.37
		Clayey mud (2.7–2.9)	3.22	3.22	3.22	0.37	0.37	0.37
		Silty clay (2.9–3.4)	5.79	5.79	5.79	1.62	1.62	1.62
		Peat (3.4–4.9)	5.89	23.90	14.89	0.62	0.74	0.68
		Sandy clay (4.9–6.4)	BDL	1.12	0.53	0.49	1.62	1.09
		Sandy & Clayey mud (6.4–7.4)	0.26	0.30	0.28	0.25	1.24	0.74
		Clayey sand (7.4–9.7)	BDL	0.12	0.05	0.37	2.86	1.72
8	Karunagapalli South	Sand (0–0.9)	0.30	0.64	0.47	BDL	0.64	0.32
		Clayey mud (0.9–1.9)	16.20	16.20	16.20	2.68	2.68	2.68
		Sand (1.9–13.5)	BDL	1.19	0.41	0.28	5.19	2.09
9	Komallur	Silty and sandy clay (0–1)	3.78	14.14	8.96	1.24	1.75	1.50
		Peat (1–2)	47.63	47.63	47.63	1.24	1.24	1.24
		Sand (2–6)	0.13	0.13	0.13	0.68	0.68	0.68
		Silty clay (6–11)	2.06	2.69	2.28	0.06	7.48	3.93
		Conglomerate (11–12.5)	Not analyzed					
10	Chunakkara	Clayey sand (0–0.4)	0.38	0.38	0.38	BDL	BDL	BDL
		Sandy clay (0.4–0.9)	0.37	0.37	0.37	BDL	BDL	BDL
		Sand (1.4–1.8)	0.54	1.11	0.82	0.12	0.15	0.13
		Clayey mud (1.8–1.9)	3.44	3.44	3.44	2.71	2.71	2.71
		Sand (1.9–5.4)	0.57	2.40	1.48	0.15	0.88	0.52
		Clayey sand (5.4–6.9)	1.22	1.22	1.22	5.30	5.30	5.30
		Silty clay (6.9–7.4)	3.26	3.26	3.26	3.86	3.86	3.86
		Clayey mud & Silty sand (7.4–9.2)	3.82	6.22	5.15	BDL	2.96	1.41
		Conglomerate (9.2–11)	Not analyzed					
11	Valummelpunja	Sand (0–3.9)	BDL	0.37	0.13	0.19	2.91	1.20
		Silty clay (3.9–4.8)	1.25	2.16	1.70	0.91	2.35	1.63
		Sand & Silty sand (4.8–5.4)	BDL	1.78	0.89	0.19	0.20	0.20
		Clayey Sand (5.4–7.9)	BDL	0.37	0.17	BDL	2.39	1.17
		Conglomerate (7.5–8)	Not analyzed					

The Total Heavy Mineral (THM) content in the sand dominant sediments of the Ayiramthengu borehole core exhibits a decreasing trend towards the bottom of the core. The content of THM varies from 3.93% to 37.39% with average of 14.41%. The top 2m of the core has high content of THM (37.39%) indicating its beach affinity. In the non-magnetite fraction of the THM, opaques (26.67–60.12%; av. 46.44%) and sillimanite (30.35–58.75%; av. 42.88%) dominates over the rest (Table 4). Small amounts of zircon, garnet and inosilicates are also reported from the area. Magnetite, rutile and biotite occur in traces in the samples. THM content of Chavara South borehole core varies from 18.35% to 24.40% with an average of 20.72%. The heavy mineral residue includes opaque as the major member (av. 87.25%; range: 82.09–91.43%); (Table 4) and, sillimanite and zircon are present as minor members, (Sillimanite: 3.09–9.95%, av. 5.24%; Zircon: 2.86–6.47%, av. 5.46%). It is axiomatic that the Ayiramthengu—Chavara stretch in the SW coast is known for economically viable deposits of beach placers. The THM content is substantially high in the Govindamuttom borehole core, especially in the middle sand dominated layer (4.73–50.17%; av. 17.51%) with the highest values at 5.5m level (50.17%). The heavy mineral species include opaque (av. 68.53%) and sillimanite (av. 27.06%) as the major members followed by zircon as minor member (0.0–9.52%; av. 2.99%); (Table 4). Rutile and monazite are present only in traces. The major mineral species in the THM contents of the other borehole cores are also similar as that of the Govindamuttom core. However, the trace minerals exhibit a wide variation. Among the trace minerals, monazite is encountered in most of the cores.

**Table 4:** Ranges and averages of total heavy minerals (in wt%) and heavy mineral species (in number %) in the subsurface sediment samples [Average is in parenthesis; THM Total Heavy Minerals].

Location	THM	Opaque	Sillimanite	Zircon	Rutile	Monazite	Garnet	Inosilicate	Biotite	Sphene
BOREHOLE CORES										
Ayiramthengu	3.93–37.39 (14.41)	26.67–60.12 (46.44)	30.35–58.75 (42.88)	2.42–6.67 (3.38)	0.0–0.75 (0.23)	0.0–6.67 (1.05)	0.0–1.78 (0.76)	0.0–1.38 (0.51)	0.0–1.67 (0.52)	0.0–2.42 (0.44)
Chavara South	18.35–24.40 (20.72)	82.09–91.43 (87.25)	3.09–9.95 (5.24)	2.86–6.47 (5.46)	0.0–0.90 (0.51)	0.0–2.26 (0.97)	-	-	-	-
Govindamuttom	4.73–50.17 (17.51)	52.35–96.94 (68.53)	2.04–43.53 (27.06)	0.0–9.52 (2.99)	0.0–1.84 (0.38)	0.0–0.71 (0.05)	-	-	-	-
Pathiyur	0.45–31.61 (9.63)	0.0–60.83 (29.77)	0.0–66.09 (36.11)	0.0–3.33 (1.80)	-	0.0–0.99 (0.25)	0.0–0.83 (0.18)	-	0.0–1.38 (0.17)	0.0–0.99 (0.27)
Vettiyar	4.08–18.22 (8.35)	51.84–84.75 (69.18)	9.04–43.26 (26.59)	1.49–4.52 (2.97)	0.0–0.41 (0.07)	0.0–0.56 (0.17)	-	-	-	-
Karunagapalli South	3.60–47.46 (31.36)	72.79–85.86 (80.38)	8.67–20.54 (13.40)	2.23–8.04 (4.53)	0.0–0.85 (0.21)	0.0–3.13 (0.56)	-	-	-	-
Chunakkara	0.49–4.08 (2.46)	21.03–98.00 (62.06)	1.50–76.38 (35.17)	0.44–4.40 (2.05)	-	-	-	-	-	-
Valummelpunja	3.50–5.84 (4.46)	31.73–48.37 (42.47)	48.91–64.66 (54.03)	1.09–3.59 (2.11)	0.0–0.54 (0.14)	0.0–0.60 (0.25)	-	-	-	-

### 4.2 Geochronology and palynology

A total of 27 dates of 15 borehole sediments (including the available published dates) have been used for establishing chronology of the sedimentary deposits. Out of the 27 dates, 16 are of Late Pleistocene and the remaining are of Holocene (Table 5). The Late Pleistocene dates vary from 46,570 ± 3480 to 16190 ± 360 yrs BP and are confined generally to the sedimentary deposits in the eastern half (wetlands and older coastal plains) of the study area. Pleistocene dates are also recorded in the lower part of some of the borehole cores in the western side (e.g. Ayiramthengu, Ramapuram). Holocene dates are recorded generally for the cores retrieved from the younger coastal plains and also some of the wood samples collected from the surface layers of the wetlands in the eastern half. The dates vary from 2460 ± 120 yrs BP to 9680 ± 120 yrs BP (Table 5). The youngest date is recorded for the vegetal remains embedded in the ridge sand buildup at Ramapuram. Muthukulam borehole core shows ^14^C dates (3662 ± 114 yrs BP, 6276 ± 112 yrs BP and 7176 ± 82 yrs BP) of Holocene age [42]. A gastropod shell from Ayiramthengu borehole at 4.75 m below ground level (bgl) was dated and found to be 2580 ± 110 yrs BP. Two carbonaceous clay samples of Pathiyur borehole from 5.0 m (40,000 yrs BP) and 9.0 m (28,830 ± 2330 yrs BP) levels gave Late Pleistocene age (Table 5). Almost identical dates have been obtained for a peat sample at 1.0 m level (20,600 ± 1030 yrs BP) and organic matter rich sediment at 10.0 m level (43,738 ± 574 yrs BP) of the Komallur borehole core. The dominance of Late Pleistocene age in most part of the borehole core indicates the role of Late Pleistocene—Early Holocene geological events in giving rise to the landform features of the study area.

**Table 5:** Radiocarbon dates of sediments, peat, wood and shells.

SI. No.	Sample Location	Lab Number	Material	Depth (m)	^14^C Dates (yrs BP)
1	Eruva ^(1)^	-	Shell	2.1–2.2	36218 ± 813
		-	Shell	4.03–4.05	39193 ± 923
		-	Shell	6.64	42490 ± 860
2	Muthukulam^(1)^	-	Wood	1.27–1.35	3662 ± 114
		-	Wood	2.07–2.12	6276 ± 112
		-	Wood	3.0–3.10	7176 ± 82
3	Pathiyur	-	Sediment	5.0	> 40000
		-	Sediment	9.0	28830 ± 2330
4	Chunakkara	BS- 3475	Sediment	1.9	33040 ± 3400
		BS- 3472	Sediment	9.0	44060 ± 190
5	Vettiyar	BS-3473	Peat	3.5	5460 ± 40
6	Komallur	-	Peat	1.0	20600 ± 1030
		-	Sediment	10.0	43738 ± 574
7	Ayiramthengu	BS- 2595	Shell	4.8	2580 ± 110
		BS- 2596	Sediment	20.4	40000
8	Valummelpunja	BS-3474	Sediment	5.0	46570 ± 3480
9	Karunagapalli	BS-3482	Sediment	6.9	7270 ± 250
10	Vayyankara chira	BS-3559	Sediment	0.3	16190 ± 360
11	Vatta kayal	BS-3560	Wood	2.0	9680 ± 120
		BS-3566	Wood	2.0	2730 ± 3 0
12	Parayakadavu^(2)^	-	Shell	6.5	4610 ± 100
13	Karipuzha^(2)^	-	Sediment	3.8	7140 ± 90
			Shell	8.5	43470 ± 1820
14	Ramapuram^(2)^	-	Wood	3.0	2460 ± 120
		-	Shell	7.6	22450±710
		-	Shell	12.0	39370 ±1000
15	Thevalakkara^(3)^	BS-2916	Sediment	11.0	41788 ± 574

1 [42]; 2 [18]; 3 [14]

**Fig 5:**
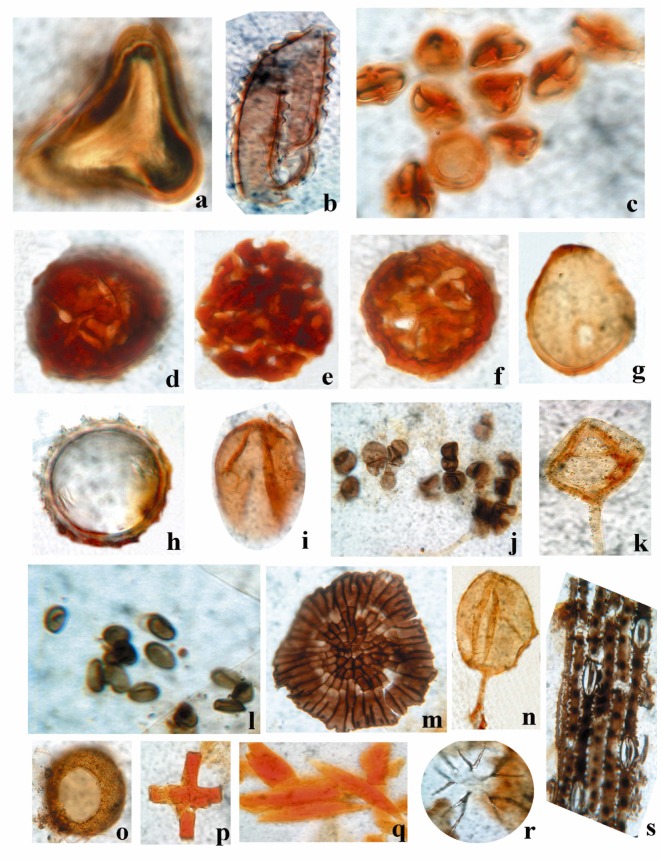
Palynological assemblage (selected forms only) of the Chunakkara and Govindamuttom borehole cores. a. Pteridophytic spore, b. *Ceratopteris* sp. (Freshwater floodplains), c. *Rhizophora sp*. (Rhizophoraceae)*—*Mangrove pollen, d, e, f. *Ctenolophonidites costatus* (Neogene reworked), g. *Cullenia exarillata–*Evergreen plant–Heavy rainfall, h. Malvaceae pollen., Gramineae pollen.j, l -Fungal spores. k, n*–Glomus* sp. (Fungal spore)–Erosion Indicator. m. Fungal fruiting body. o. Thecamoebian cyst. p, q. Sponge spicules. r. *Staurastrum* sp. s. Gramineae cuticle.

**Fig 6:**
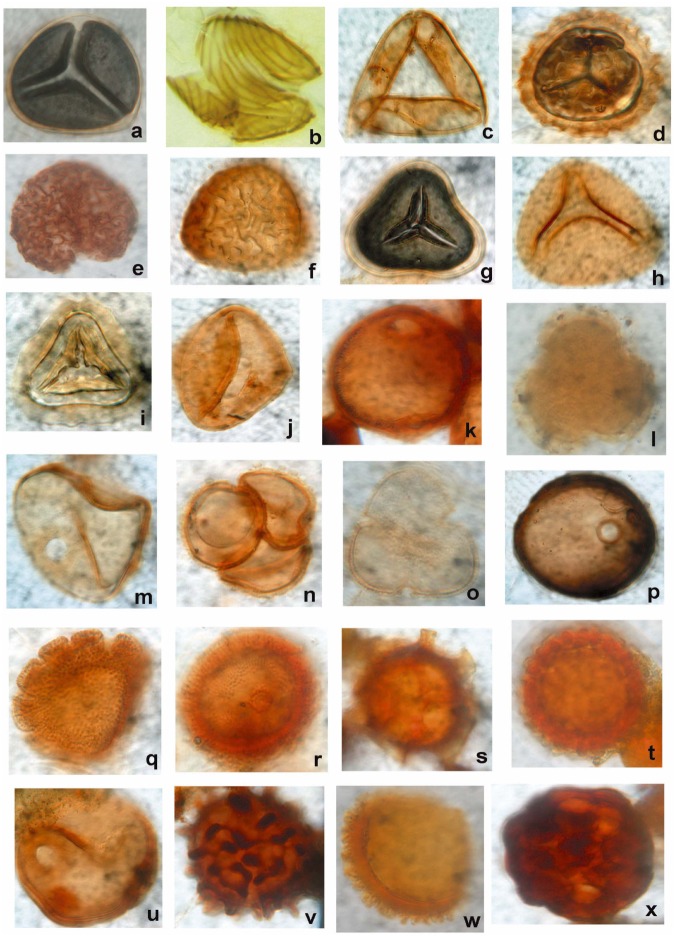
Palynological assemblage (selected forms only) of Vettiyar, Karunagapalli South and Valummelpunja borehole cores. a. *Cyathea* sp. (Cyatheaceae)–[Karunagapalli 90–100]. b. *Ceratopteris thalictroides–*Flood plain indicator [Karunagapalli 90–100]. c. *Gleichenia* sp.*–*Trilete fern spore [Karunagapalli 90–100]. d, g & i. *Pteris* sp.–High humidity [Karunagapalli 90–100]. e. *Lycopodium* sp. [Karunagapalli 90–100]. f. *Lygodium* sp. [Karunagapalli 90–100]. h. *Todisporites flavatus (Osmundaceae)* [Karunagapalli 90–100]. j, k, m, u. *Cullenia* sp.–Heavy rainfall indicator [Karunagapalli 90–100, Karunagapalli 690–700, Karunagapalli 690–700, Valummelpunja 740–750 respectively]. l. Caesapiniaceae pollen—[Karunagapalli 1340–1350]. n, o. *Droseridites* sp. [Karunagapalli 690–700]. p. *Lakiapollis* sp.*–*Modern analogue of *Cullenia* sp. [Vettiyar 960–970]. q, r, s,t,v,w,x–*Ctenolophonodites* sp.

The topmost part of the Ayiramthengu borehole core gave Holocene (2,580 ± 110 yrs BP) age whereas the bottom most part showed Late Pleistocene age (40,000 yrs BP); (Table 5). In the Ayiramthengu core, the algal palynomorphs are represented mainly by cyanobacteria. Marine representatives such as dinoflagellates and foraminiferal linings are frequently found in a few intervals of the profile as the location site is not very far from the intertidal zone. Except for a short interval at 4.0–7.0 m, cyanobacteria are well represented in the rest of the borehole core. Abundance of cyanobacteria at 12.0 m and 19.0 m levels are probably attributed to heavy rainfall and in fact, these are the only intervals showing wet periods of the Early to Middle Holocene. The interval between 20.4 and 21.0 m displays a progressive decrease in cyanobacterial population. Presence of *Ceratopteris* spores, thecamoeba, sponge spicules and Gramineae pollen in the borehole cores indicates terrestrial input whereas the presence of foraminiferal linings and dinoflagellates indicate marine influence at this level. Palynological examination of Govindamuttom borehole core, the top 0.9–1.0 m layer, comprises sponge spicules, *Staurastrum* sp., Gramineae pollen and Thecamoeba, *Ceratopteris* sp. etc (Table 6). The organic recovery was poor in the middle layer (5.4 to 5.5m). However, presence of a few sponge spicules and fungal bodies are detected in the palynological preparation. The bottom layer at 13.9–14.0 m below ground level (bgl) comprises dominance of *Botryococcus*, Thecamoeba and sponge spicules. The entire sequence is formed under marine process, but later subjected to aeolian and fluvial activities. Figs 5 and 6 show a few representative palynomorphs in the studied borehole cores. In Pathiyur borehole core, the lowermost lithological unit at 12.9–13.0 m bgl registers low organic matter content. The sediment at a depth 9.0–12.0 m bgl shows dominance of structural terrestrial remains and microscopic charcoal (Table 6). Presence of pollen, cuticles, fungal complex, dinocyst, foraminiferal linings, dinoflagellates, cyanobacteria, sponge spicules and scolecodonts indicates terrestrial freshwater environment with marine influence. Above this layer, presence of foraminiferal linings, pollen, diatom, fungal complex, dinoflagellates and structural terrestrial remains has been observed. This shows the prevalence of shallow marine / intertidal environment during the deposition of sediments at this level. The shallow marine environment continued up to 5.0 m and above that, presence of structural terrestrial remains, microscopic charcoal and the thecamoeba is common indicating terrestrial freshwater environment with higher acidity and / or pollution (Table 6).

**Table 6:** Sediment characteristics and palynological observations of the borehole cores.

Borehole location	Depth (m)/ ^14^C dates	Sediment characteristics	Palynological observations	Remarks
**Ayiramthengu**	0.9–1.0	Yellowish sand with mica	Foraminiferal lining are abundant. *Ceratopteris* and pteridophytic spores present. Thecamoeba and *Discoaster* seen.	Marine facies with fresh water influx; facies change towards freshwater indicated by *Ceratopteris* and also level of pollution due to Thecamoeba.
	9.9–10.0	Yellowish brown sand with mica	Pteridophytic spores abundant. This is followed by the Thecamoeba and *Discoaster* few. Presence of *Cullenia* pollen & tricolpate pollen grain.	Suggestive of stressed terrestrial environment with slight marine influence and at the same time rainfall seems to be fair at this level.
	10.4–10.5	Yellowish brown black clay hard	Pteridophytic spores few followed by thecamoebians; foraminiferal linings are few; *Meliolinites* found to be present.	Precipitation and strong terrestrial input indicate wet conditions & higher atmospheric pressure at this level.
	13.0–14.0	Grey black sand	*Ceratopteris* spores dominant. *Rivularia* follows this; foraminiferal linings and tests few; dinoflagellate cyst, thecamoeba, charcoal fragments, fungal fruiting bodies, hyphae also found.	Transitional facies suggests fresh water floodplain and eventual exposure of wetland for considerable period with occasional tidal inputs.
	17.9–18.0	Greyish green clay	*Rivularia* few but more than foraminiferal linings. Malvaceae pollen, *Ceratopteris* spores few. Thecamoeba, dinoflagellate cyst also present. Gramineae pollen found.	Facies change is indicated here as the brackish water getting more influence of tidal waves.
	18.5	Grey clay	Pteridophytic spores-*Pteris*, *Ceratopteris* abundant. This is followed by *Cullenia* pollen. Few foraminiferal lining and fungal fruiting bodies found to be present. *Rivularia* forms also present; *Euphorbiaceae* pollen also seen.	Dominant fresh water facies. However, tidal influence seen by the presence of foraminiferal linings. Strong terrestrial and presence of ever green taxa indicative of good rainfall and wet climate.
	18.9–19.0	Grey clay	*Ceratopteris* spore dominant along with other kind of spores (Pteridophytic). Few fungal fruiting bodies found to be present. Foraminiferal linings few. *Rivularia* also found to be present. Dinoflagellates present. Fungal spores few.	This level is more or less same as above.
	20.4–20.5	Black clay	Pteridophytic spores dominant. *Rivularia* few, dinoflagellates few; Presence of salt gland, charcoal. Foraminiferal linings of different type showing constriction in between two lobes. Dinoflagellates few; *Ceratopteris* spores dominant.	Humic conditions and prevalence of wet climate; marine influence is also seen may be strong/ higher sea level?
	22.9–23	Brown mud	Organic material without any known type of microfossil.	Less sedimentation and low input either from terrestrial/ aquatic sources. Quiscent period? Dry/Arid conditions
	25.5	Brown hard mud layer	Thecamoeba present. Malvaceae pollen. Pteridophytic spores, Discoaster few.	Fresh water facies prevalent and may be exposed really for a considerable period of time.
**Govindamuttom**	0.9–1.0	Greyish white, medium to fine sand	Abundance of fungal spores followed by Sponge spicules; *Staurastrum* few; Gramineae pollen and cuticles; charcoal, thecamoeba and *Ceratopteris* spores few.	Freshwater; humid conditions; heavy rainfall.
	5.4–5.5	Greyish white, medium to fine sand	Organic recovery poor; few sponge spicules, Bacteriastrum and fungal fruiting bodies.	Dry period with slight marine input.
	13.9–14.0	Greyish black, clay dominated sediments	Dominance of *Botryococcus* followed by sponge spicules and thecamoeba.	Dry period with increased levels of pollution, acidic and stress conditions.
**Pathiyur**	1.9–2.0	Light grey, medium to fine sand	Presence and dominance of structural terrestrial remains and microscopic charcoal, presence of thecamoeba.	Terrestrial fresh water environment with acidity or pollution.
	2.9–3.0	Light grey, medium to fine sand	Presence and dominance of structural terrestrial remain. Presence of dinocyst & insect remains.	Terrestrial environment with marine influence.
	4.3–4.4	Sandy mud	Presence and dominance of structural terrestrial remain & microscopic charcoal. Presence of pollen and spore.	Terrestrial environment.
	5.3–5.5	Sandy mud	Presence and dominance of foraminiferal lining. Presence of pollen, diatom, fungal complex, dinoflagellate & structural terrestrial remains.	Marine environment.
	5.9–6.0	Greyish green, stiff clay with shell dust	Presence and dominance of foraminiferal lining. Presence of cyanobacteria, insect remain, structural terrestrial remain & microscopic charcoal.	Marine environment.
	6.4–6.5	Greyish green, stiff clay	Presence and dominance of structural terrestrial remain & microscopic charcoal. Presence of pollen, foraminiferal lining, dinoflagellate, cyanobacteria, insect remains & sponge spicule.	Terrestrial fresh water environment with marine influence.
	7.9–8.0	Greyish green, sandy mud	Presence and dominance of foraminiferal lining. Presence of pollen, dinocyst, cyanobacteria, structural terrestrial remain and microscopic charcoal.	Marine environment.
	8.9–9.0	Greyish black, sandy mud	Presence and dominance of structural terrestrial remain & microscopic charcoal. Presence of pollen, leaf cuticle, fugal complex, dinocyst, foraminiferal lining, dinoflagellate, cyanobacteria, *Bacteriastrum*, sponge spicule & scolecodonts.	Terrestrial freshwater environment with marine influence.
	10.9–11.0	White, coarse to medium sand	Presence and dominance of structural terrestrial remains & microscopic charcoal. Presence of fungal complex, dinocyst, *Bacteriastrum*, dinoflagellate, foraminiferal lining, sponge spicule & scolecodonts.	Terrestrial environment with marine influence.
	11.9–12.0	Stiff, clayey sand	Presence and dominance of structural terrestrial remain. Presence of sponge spicule.	Terrestrial environment with marine influence.
	12.9–13.0	Silty sand	Poor recovery of organic matter.	Poor recovery of organic matter.
**Vettiyar**	2.2–2.3	Greyish brown, silty clay	Organic recovery is very high; dominated by reworked pollen of *Ctenolophonidites* sp. The pollen shows high organic maturation and resembles to that of Warkalli Formation; charcoal particles in the form of charred cuticles of grasses frequent, indicating fire activity; a few pteridophytic spores are also observed.	Mainly continental/ fresh water facies; evidence of erosional /reworking of Neogene sediments as the organic particles with high maturation level.
	4.9–5.0	Peat	Very good organic recovery and assemblage dominated by fungal spores; few pteridophytic spores too occur; reworked Neogene pollen *Ctenolophonidites* sp., occasionally seen.	Continental facies, mainly freshwater, sediments are of reworked type as the organic matter show heavy maturation; prevalence of high humidity due to fungal spores.
	9.6–9.7	Reddish brown, clayey sand	Relatively poor organic recovery; non-pollen forms are seen; *Lakiapollis* sp. (resembling modern *Cullenia exarillata*) of Neogene observed.	Probably fluctuating facies as a few elements of shallow coastal/tidal elements (*Bacteriastrum)* seen besides some desmids; exposure of the sedimentary facies envisaged; erosion/reworking indicated due to Neogene pollen.
**Karunagapalli South**	0.9–1.0	Greyish black, clayey mud	Organic recovery is rich; dominance of pteridophytic spores (*Ceratopteris thalictroides*, *Pteris* sp., *Lycopodium* sp., *Lygodium* sp., *Gleichenia* sp., *Osmunda* sp.), reworked Neogene pollen *Ctenolophonidites* sp. and *Cullenia* sp. Abundance of fungal remains.	Erosional activity; High humidity. Fresh water facies and flood plain.
	6.9–7.0 **(7,270± 250 yrs BP)**	Light grey, medium to fine sand	Organic rich, abundance of pteridophytic spores and fungal spores and hyphae (*Diporisporites* sp. *Pluricellaesporites* sp, *Multicellaesporites*, *Meliolinites* sp); Pollen of *Drosera* and spore of *Ceratopteris* sp. frequent; Few types of plankton (*Bacteriastrum* sp.)	Freshwater and flood plain facies and probable fluctuating tidal environment;? Reworked Neogene sediments indicate heavy rainfall and erosion.
	13.4–13.5	Yellowish, medium to coarse sand	Comparatively poor organic recovery; Caesalpiniaceae pollen observed; Sponge spicules/ desmids and fungal spores frequently found.	Freshwater facies and exposed sedimentary environment.
**Komallur**	0.0–0.3	Greyish black, clay dominated sediments	Dominance of structural terrestrial remains along with pollen and cuticle; cyanobacteria, microscopic charcoal and spores.	Terrestrial environment.
	1.0–2.0 **(20,600±1030 yrs BP)**	Decayed vegetal remains and buried wood	There is dominance of structural terrestrial remains. Presence of fungal complex (*Glomus* spore) and leaf cuticle, microscopic charcoal, pollen, leaf cuticle, spore and oil forming green algae *Botryococcus*.	Terrestrial freshwater environment.
	4.0–6.0	Light grey, medium to fine sand	No organic matter.	No organic matter suggesting an ecological shift.
	6.5–11.0 (43738± 574 yrs BP)	Greyish green, silty clay with broken shells	Dominance of the sponge spicule. Presence of foraminiferal lining, algae *Botryococcus* and diatoms, pollen, cyanobacteria and microscopic charcoal.	Indicate aquatic origin (marine environment).
**Chunakkara**	0.0–0.5	Yellowish brown, sandy clay with stains of iron oxides over coarser grains	Dominance of *Ctenolophonidites costatus* (reworked Neogene); few sponge spicules, *Glomus* sp., and Malvaceae pollen.	Heavy erosion and heavy rainfall.
	5.4–5.5	Light greyish green, medium to fine sand with intercalations of clay	Few Mangrove pollen (Rhizophoraceae), fungal fruiting bodies, pteridophytic spores, *Cullenia* sp and fungal hyphae.	Coastal environment with terrestrial input, likely under heavy rainfall.
	9.9–10.0	Yellowish red, conglomerate	Sponge spicules dominant.	Dry period.
**Valummelpunja**	1.8–1.9	Greyish white, medium to fine sand	Organic recovery is moderate;? reworked Neogene pollen *Ctenolophonidites* sp. and fungal spores (*Multicellaesporites* sp.).	Continental / Fresh water facies; Heavy rainfall and erosion of Neogene sediments as the palynological assemblage is a reworked one.
	4.8–4.9 (46570± 3480 yrs BP)	Greyish black, silty clay	? Reworked Neogene pollen. *Ctenolophonidites* sp. and *Cullenia* sp. Few fungal spores and hyphae (*Meliolinites* sp); pteridophytic spore (*Pteris* sp and *Cyathea* sp.); NPP types (Planktons–*Staurastrum* sp, *Bacteriastrum* sp.	Dominated by continental facies but tidal influence observed due to a few planktons; Heavy rainfall and erosion of Neogene sediments due to reworked Warkalli elements.
	7.4–7.5	Greyish white, clayey sand with pebbles	? Reworked Neogene pollen *Ctenolophonidites* sp. and *Cullenia* sp. Few fungal spores and a few planktons (*Staurastrum* sp.).	Mainly continental / fresh water facies; Heavy rainfall and erosion of Neogene sediments as reworked pollen are dominant.

In the Vayyankarachira core, the top two samples (0.1–0.2 m & 0.2–0.4 m) contain abundance of terrestrial organic matter having high organic maturation value (dark yellow to brown) with a few pale yellow coloured elements of less maturation. Both these levels contain abundance of desmids of very high maturation level (Table 6). Earlier studies revealed that desmids used to be frequently seen in the contact of Neogene-Quaternary boundaries of South Kerala Sedimentary Basin (SKSB); [20]. The abundance of desmids indicates a change in ecological facies and the sedimentary basin remained exposed for a longer period of time otherwise this much accumulation in the basin could not be envisaged. Further, both the levels have reworked palynological contents (*Ctenolophonidites*, *Malvacearumpollis*, *Protoperidinium* cysts, pteridophyte spores and *Botryococcus*) derived from Warkalli Formation. The terrestrial organic matter having much less organic maturation are mainly of Liliaceae pollen along with a few thecamoebians and as such they may be of Late Quaternary (Late Pleistocene- Holocene) period. The occurrence of these elements indicates that the area was swampy / marshy and also got exposed for a considerably long period with higher acidic level. Thecamoebian accumulation is possible only in stressed environment especially of high acidic nature and they must have been deposited during the dry period of Late Pleistocene/Holocene (?). The retrieved organic matter from all the three levels are not of Quaternary age as the organic maturation level is considerably higher although lighter coloured organic matter do occur in sparse quantity. In the Vettiyar borehole core, organic recovery is very high at 2.2–2.3m level. Palynological assemblage dominated by reworked pollen of *Ctenolophonidites* sp., shows high organic maturation level and resembles the pollen assemblage of Warkalli Formation. Charcoal particles in the form of charred cuticles of grasses, common in the samples, indicate fire activity. The peaty layer at 5.0 m level shows dominance of fungal spores (Table 6). A few pteridophytic spores and reworked Neogene pollen *Ctenolophonidites* sp. are also occasionally seen in the palynological preparation. At 10.0 m level, organic recovery is poor and the assemblage is dominated by fungal spores. A few pteridophytic spores are also identified in the samples. The top grayish black mud dominated layer of the Karunagapalli South borehole core records high organic recovery with dominance of pteridophytic spores (*Ceratopteris thalictroides*, *Pteris* sp., *Lycopodium* sp., *Lygodium* sp., *Gleichenia* sp., *Osmunda* sp.), reworked Neogene pollen *Ctenolophonidites* sp., *Cullenia* sp. and few fungal remains. The medium to fine sand layer at 7.0 m level also records high organic recovery showing abundance of pteridophytic spores, fungal spores and hyphae (*Diporisporites* sp., *Pluricellaesporites* sp., *Multicellaesporites*, *Meliolinites* sp.). Pollen of *Drosera* sp. and spore of *Ceratopteris* sp. are frequent in the sample. A few planktons (*Staurastrum* sp.) are also noticed in the sample. The bottom sand dominated layer (at 13.5m level) records comparatively poor organic recovery and contains Caesalpeniaceae pollen, sponge spicules/ desmids and fungal spores (Table 6).

The palynological assemblage of the Chunakkara borehole core exhibits a different suite of palynomorphs. The top layer (0.4–0.5 m bgl) shows dominance of *Ctenolophonidites costatus* (a Neogene reworked palynomorph), a few sponge spicules, *Glomus* sp. and Malvaceae pollen (Table 6). Heavy erosion in the hinterlands under extreme rainfall events might be the reason for the observed suite of palynomorphs in the borehole sample. The middle layer (5.4–5.5 m) is composed of a few mangrove pollen represented by Rhizophoraceae, fungal fruiting bodies, *Cullenia* sp., and fungal hyphae. The bottom layer (9.9–10 m) is composed essentially of sponge spicules (Fig 5). In the Komallur borehole core, the top clay rich layer indicates dominance of structural terrestrial remains along with pollen and leaf cuticle (Table 6). Presence of cyanobacteria, microscopic charcoal, fungal complex is also noticed in this layer. The bottom part of the layer records oil forming green algae, *Botryococcus*. The sand dominant layer below this does not contain any organic matter, suggesting an ecological shift in the depositional facies. Below this, is a layer formed under marine environment. It is characterized by dominance of sponge spicule, foraminiferal lining, diatom, algae *Botryococcus*, cyanobacteria and microscopic charcoal. In the Valummelpunja borehole core, the top sand layer (at 2.0 m level) records moderate recovery of organic matter with reworked Neogene pollen, *Ctenolophonidites* sp., and fungal spores (*Multicellaesporites* sp.). The silty clay layer at the middle 5.0m level shows dominance of reworked Neogene pollen, *Ctenolophonidites* sp., and *Cullenia* sp., few fungal spores and hyphae (*Meliolinites* sp.). Pteridophytic spores are also noticed in this layer. The bottom, pebble dominated layer at 7.5–8.0 m level is dominated by reworked Neogene pollen of *Ctenolophonidites* sp., and *Cullenia* sp. (Fig 6). A few fungal spores and plankton (*Staurastrum* sp.) are also detected in the core (Table 6).

## 5. Discussion

Many factors including sea level oscillations and climate changes have played a pivotal role in shaping of the coastal lands. Like many of the world’s coastlines, the Indian coastline too has been subjected to coastal processes and terrestrial modifications over the years. The varied geo-environmental conditions prevailing in the coastal lands have resulted in the formation of a wide range of geomorphological features. The coastline has oscillated many times due to marine transgression and regression in the Quaternary period. The transgression has enhanced the aerial spread of the sea submerging a greater part of the land under saline waters. Coastal deposition became extensive during the regressive phases. On gently-shelving coast, sand brought from the sea floor got deposited as beaches, spits and barriers. In the river mouths, fluvial sediments accumulated as deltas, and in sheltered bays and estuaries muddy sediments accreted in the form of mudflats and / or mangrove swamps. Thus, the change in sea level at a given location of the coastal area has been the result of the combined effects of global climate change and of local / regional scale landform changes. Major part of the coastal plains are covered by water bodies such as lagoons and perennial/seasonal wetlands with lagoonal deltas and fans and the rest is made up of the ridge-runnel system formed by Holocene geological events [43]. As of now the subsurface sediments of coastal stretch along southwestern India have provided ample evidence to decode the sea level oscillations and climate variability during the Late Quaternary [6, 9, 10, 14, 19, 20, 28, 44–46]. The accrued data has been found to be significant while addressing the geomorphic response to landform dynamics and proposing a terrain evolution model for the coastal land subjected to the present study. Climate variability, palaeoenvironment, drainage pattern of rivers associated with the coastal plains, subsurface stratigraphy and coastal dynamics have been integrated while addressing geomorphic evolution of the Late Quaternary period.

### 5.1 Late Quaternary climate change and sediment architecture

**Fig 7:**
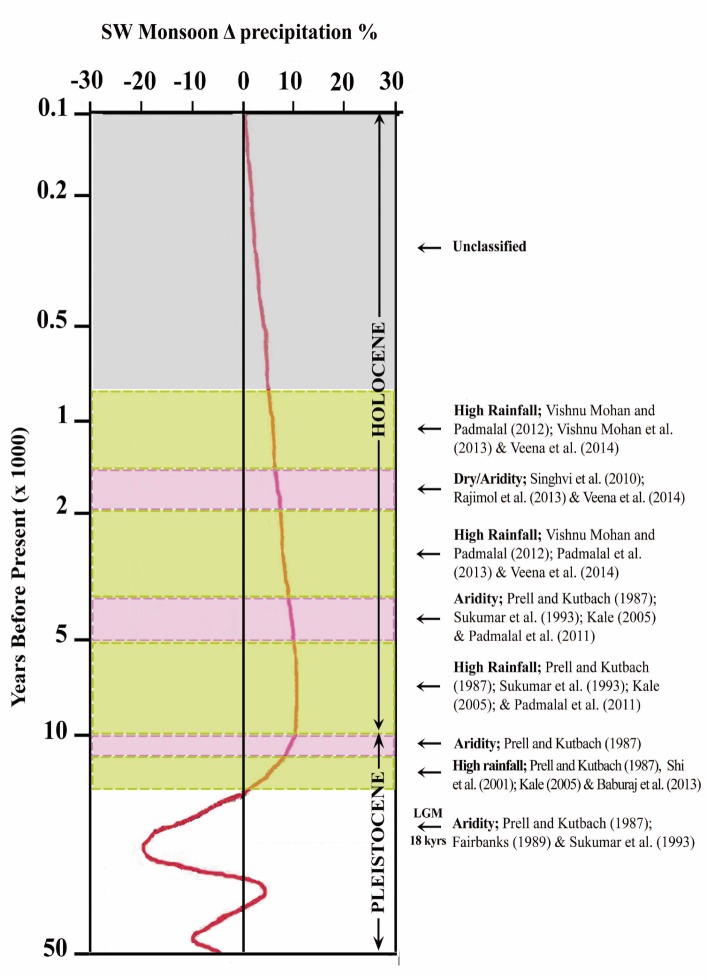
Palaeoclimate reconstruction of the Late Quaternary, specifically post LGM period in SW India derived from data of the present study and published works.

Analysis of climate proxies of the last 20,000 years accrued from drilled core sediments of the present study together with the published results [47–54] revealed that the study area has experienced dramatic changes of climate in the post LGM period. Fairbanks [36] revealed that during LGM (i.e., around 18 k yrs BP), the sea level was approximately 120.0 m lower than what it is now due to cold and dry climatic conditions. Later, the sea level rose to about 20.0 m during the period 17,100–12,500 yrs BP which is marked as the first phase of deglaciation. This phase was terminated by a rapid sea level rise of 24.0 m in less than 1000 years. The formation of peat at the Komallur wetland with an age of 20,600 ± 1,030 yrs BP coincides with the regressive phase [51] of LGM. Palynological / micropalaeontological analysis reveals that these palaeo-wetlands are evolved presumably due to the land—sea interactions of the last interglacial prior to Late Pleistocene [7, 14, 19]. Occurrence of Late Pleistocene deposits were also reported from the shorelines along the west coast of India [55] and from the coastal lands of Rameswaram [1]. The radiocarbon dates of Late Pleistocene deposits of the present study area are generally in the range of 20,600 ± 1,030 yrs BP and 46,570 ± 3,480 yrs BP. The medium to fine grained, well sorted quartzose sand lying directly above the greenish grey clay in the Komallur borehole core is nothing but the beach/ littoral sediments formed under regressive phase. This was followed by the development of peat and clay apron under terrestrial freshwater regime. The palynological contents, especially the presence of *Cullenia exarillata*, are an indication of heavy rainfall and wet climate during Late Pleistocene. The heavy rainfall is also well recorded in the Early Holocene sediments of the study area and Konkan along SW coast of India [46, 56]. The organic rich sediments in the wetlands and ^14^C dates of 9,680 ± 120 yrs BP for the wood sample at Vatta *kayal* and 7,176 ± 82 yrs BP for the wood sample at Muthukulam together with our earlier studies in the Ashtamudi and Paravur basins [9, 14] reveal that the palaeoclimate of the Early Holocene was favorable for dense growth of forest vegetation in the area. Fig 7 shows the climate model deduced from the results of the present study as well as other published works.

The thickness of the Quaternary sediments observed in the study location varies from a few meters to over 35.0 m. The coastal lands of the study area, south of the Ashtamudi estuary are drained by many coastal plain rivers [57]. With the exception of the Pallikkal River which is a lowland river, small rivers especially in the category of coastal plain rivers (rivers originating from head water elevation < 100m amsl) are absent in the areas north of Ashtamudi Lake [58]. Furthermore, the area is subjected to severe coastal erosion and cliff retreat during Late Pleistocene which is evident from the presence of extensive coastal plain sand deposition in the area. According to Narayana and Priju [27] geologic / geomorphic evolution of coastal land hosting valleys and wetlands was influenced by many local/regional factors like changes in climate, sea level and local and regional tectonics. The chain of wetlands in the eastern peripheral zones of the study area is marked by the occurrence of Late Pleistocene sediments. These basins received eroded sediments from the Neogene hillocks that formed the hinterland areas of the Late Quaternary basin. The presence of *Ctenolophonidites costatus*, the Neogene reworked palynomorphs in the palynological contents reiterates this view. Palynological data suggests that the major provenance of the Late Pleistocene sediments in the wetlands was the hinterlands in its eastern side. Stratigraphically, the area is composed mainly of Neogene clays, calcareous beds / limestone and Archaean crystallines. The sediments in the inland wetlands on the east give indications of transgressive–regressive sequence with marine sediments at the base followed upwards by transitional and fresh water environments. Sediments of marine and transitional environments have been observed in all locations, except at Vettiyar and Karunagapalli borehole sites, where Holocene sediments unconformably overlie the Neogene deposits.

**Fig 8:**
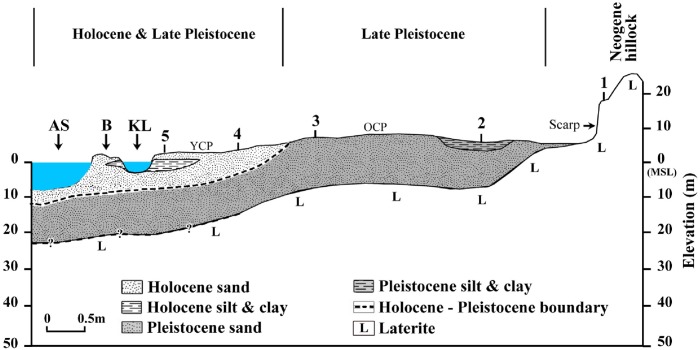
A transect along Vayyankarachira showing the Quaternary sediments (1) Komallur (2) Pathiyur (3) Ramapuram and (4)Muthukulam borehole locations. AS Arabian Sea; KL Kayamkulam Lagoon; B Beach; YCP Younger Coastal Plain; OCP Older Coastal Plain.

**Fig 9:**
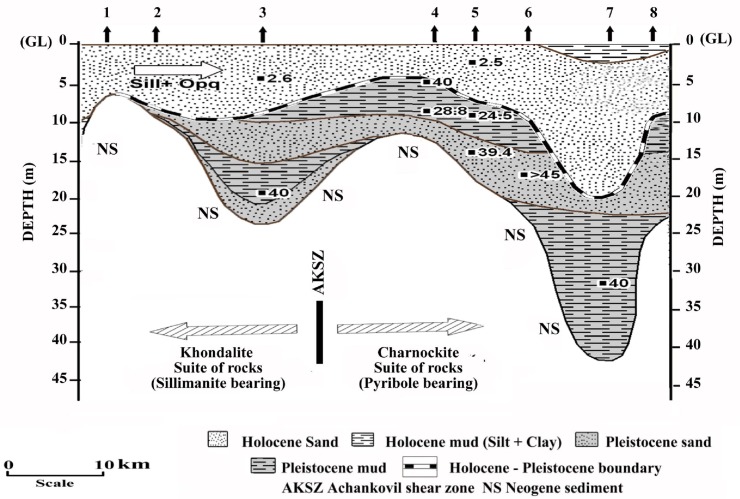
Subsurface stratigraphy of a transect along Chavara—(1) Ponmana—(2) Ayiramthengu—(3) Pathiyur—(4) Ramapuram—(5) Haripad—(6) Thottapally—(7) and Thakazhi—(8) borehole locations.

Fig 8 depicts the E—W section through Vayyankarachira and Pathiyur, Ramapuram and Muthukulam borehole locations showing the subsurface scenario of the sedimentary archives of Late Pleistocene–Holocene period and its probable boundary. The eastern half of the section is dominated by Late Pleistocene sediments, whereas the western counter parts by Holocene sediments. The N—S section, on the other hand, shows the occurrence of Late Pleistocene deposits in its lower part and Holocene sediments in its upper part. This clearly indicates that the geomorphic features of this area have been evolved due to the combined effects of Late Pleistocene and Holocene coastal processes as shown in the subsurface stratigraphy of the investigated block (Fig 9). The inland wetlands of the study area appear to have developed as a result of Late Pleistocene sea level rise during the period 30–50 k yrs BP. There have been several episodes of sea level rise during this time. All these episodes are obviously not recorded in the sedimentary archives in the locations studied. It is interesting and also intriguing that the inland wetlands contain the records of sea level rise. It appears almost certain that the present coast in several stretches has undergone uplift and erosion, and loss of its sedimentary records.

The borehole cores of the inland wetlands reveal unique features. One of them is Komallur core in which a typical a regressive sequence that subsequently ends up with a freshwater swamp facies. The formation of peat having an age of 20,600 ± 1,030 yrs BP coincides with the regressive phase which attained its maximum around 18,000 yrs BP. Palynological / micropalaeontological analysis of the lower greyish green silty clay reveals that the wetlands are evolved due to the land-sea interactions took place around 40–30 k yrs BP. The medium to fine grained, well sorted quartzose sand lying directly above the greenish grey clay represents the beach / littoral sediments formed under the regressive phase. This was followed by the development of peat and clay apron evolved under continental freshwater environments. Lithological characteristics (Fig 4) and body fossils of pelecypods and gastropods of Pathiyur and Ramapuram borehole cores reiterates the prevalence of Late Pleistocene sea which was in existence until 24,450 ± 710 yrs BP at Ramapuram. The basal unit of the Late Pleistocene sediments at Komallur and Pathiyur are made up of hard laterites/duricrusts. However, at Ramapuram the unit is represented by white clayey sands. The palynological contents, especially the presence of *Cullenia exarillata* is an indication of heavy rainfall and wet climate during Late Pleistocene. The observation is in agreement with the recent report of Kumaran et al. [59] from Konkan coast in SW India and the earlier report of Shi et al [60] who inferred that the period 40–28 k yrs BP experienced high monsoon rainfall in the Asian continents. In short, the present study reveals that the chain of coastal wetlands that are seen in the eastern periphery of the old coastal plains of the study area have been carved into distinct landforms during Late Pleistocene higher sea levels.

### 5.2 Evolution of the coastal lands and proposed terrain evolution model

**Fig 10:**
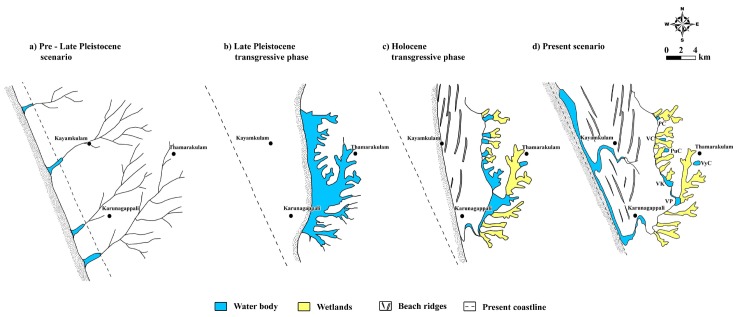
Different stages in the evolutionary history of the coastal lands between Achankovil and Thenmala Shear zones. a) Pre—Late Pleistocene scenario. Note the coastal plain rivers of the Pre–Late Pleistocene cliffed coast; b) Late Pleistocene transgressive phase. The coastal plain rivers disappeared due to denudation and cliff retreat. The digitations in the eastern border indicate the locations of the upper feeder channels of the coastal plain rivers. Valleys of the feeder channels are bordered due to tidal inundation; c) Late Pleistocene regressive phase showing the development of NNE-SSW trending beach ridges. d) Present scenario showing younger coast parallel beach ridges (NNW-SSE trending) that are making an angle with the older NNE- SSW trending beach ridges. PC Puvathuchira; VC Vallikkunnathuchira; PuC Puduchira; VyC Vayyankarachira; VK Vatta *kayal*; VP Valummelpunja.

The climate variability data (Fig 7), analysis of coastal drainage characteristics, coastal dynamics of the East–West transect along Vayyankarachira (Fig 8) and subsurface stratigraphy of East-West transect between Chavara and Thakazhi (Fig 9) have been enough for decoding the Late Pleistocene and Holocene coastal processes and the subsequent development of various geomorphic units in the study area. The landform dynamics in response to sedimentation pattern and development of various geomorphic units prior to Late Pleistocene to the present has been provided in the form a terrain evolution model (Fig 10). An evaluation of the coastal plain rivers in the uplifted block south of Achankovil Shear Zone (ASZ) reveals that the cliffed coast south of the Ashtamudi estuary hosts many coastal plain rivers [58]. Surprisingly, such rivers are absent in the coastal lowlands of the study area north of Pallikkal River. However, careful examination of the geomorphic signatures reveals that the inland wetlands in the coastal lands of the study area are nothing but the remnants of the valley floors of the upper drainage channels of coastal plain rivers that existed during the Late Quaternary period, certainly prior to the Late Pleistocene. The Late Pleistocene transgressive events might have leveled down a major portion of the land areas drained by the coastal plain rivers to Mean Sea Level. The NNE—SSW trending beach ridges located close to the inland wetlands are indicative of the extent of the Late Pleistocene transgression to which the region has been subjected to. The younger coast (present) parallel beach ridges on the other hand, indicate the extent of the Mid Holocene transgressive phase. The proposed schematic model (Fig 10) depicts the evolutionary stages of the coastal lands of the study area, especially between Achankovil and Thenmala Shear zones in Trivandrum block. The zone of convergence of the two sets of beach ridges coincides with the areas of economically viable heavy mineral placers. This clearly indicates that the heavy minerals derived from the breakdown/coastal retreat of the uplifted Neogene deposits underwent size and density based sorting at least in two transgressive phases—the Late Pleistocene and Middle Holocene. This is perhaps, the reason for the workable level of segregation of beach placers in the Neendakara—Chavara and nearby areas of SW coast of India.

## 6. Conclusions

The sediment archives retrieved from the subsurface coastal deposits of the uplifted block of South Kerala Sedimentary Basin between Achankovil and Thenmala Shear zones provided the geomorphic signatures attributed to sea level oscillations, climate variability and neotectonic activities during the Late Quaternary. These signatures are found to be useful and helped decoding the landform dynamics and addressing aspects related to the coastal processes. The sediment archives and the preserved proxies contained in them along with geochronological data indicate that the inland wetlands in the designated coastal plains are essentially the broadened remnants of the upper drainages of their pre-existed coastal plain rivers of post-Neogene age but certainly prior to Late Pleistocene. The proposed terrain evolutionary model developed from the study shows that the Late Pleistocene transgressive events might have carved out a major portion of the land areas drained by the coastal plain rivers. The NNE—SSW trending beach ridges located close to the inland wetlands indicate the extent of transgression to which the region has been subjected to during Late Pleistocene period. The present beach parallel ridges in the younger coastal plain reveal the extent of the Mid Holocene transgression. The zone of convergence of the two sets of beach ridges coincides with the areas of economically viable heavy mineral placers that are segregated due to repeated size and density based sorting during the transgressive phases.
